# Cristae architecture is determined by an interplay of the MICOS complex and the F_1_F_O_ ATP synthase via Mic27 and Mic10

**DOI:** 10.15698/mic2017.08.585

**Published:** 2017-07-20

**Authors:** Katharina Eydt, Karen M. Davies, Christina Behrendt, Ilka Wittig, Andreas S. Reichert

**Affiliations:** 1 Cluster of Excellence Macromolecular Complexes, Goethe University Frankfurt, Max-von-Laue-Str. 15, Frankfurt am Main, Germany.; 2 Mitochondrial Biology, Buchmann Institute of Molecular Life Sciences, Goethe University Frankfurt, Max-von-Laue-Str. 15, 60438 Frankfurt am Main, Germany.; 3 Department of Structural Biology, Max Planck Institute of Biophysics, Max-von-Laue Str. 3, 60438 Frankfurt am Main, Germany. Present address: Molecular Biophysics and Integrative Bio-Imaging Division, Lawrence Berkeley National Laboratory, Berkeley, CA 94720.; 4 Institute of Biochemistry and Molecular Biology I, Medical Faculty Heinrich Heine University, Universitätsstr. 1, 40225 Düsseldorf, Germany.; 5 Functional Proteomics, SFB 815 Core Unit, Faculty of Medicine, Goethe University Frankfurt, Theodor-Stern-Kai 7, 60590 Frankfurt am Main, Germany.

**Keywords:** membrane structure, bioenergetics, mitochondria, cristae, membrane protein complex, crista junction

## Abstract

The inner boundary and the cristae membrane are connected by pore-like structures termed crista junctions (CJs). The MICOS complex is required for CJ formation and enriched at CJs. Here, we address the roles of the MICOS subunits Mic27 and Mic10. We observe a positive genetic interaction between Mic27 and Mic60 and deletion of Mic27 results in impaired formation of CJs and altered cristae membrane curvature. Mic27 acts in an antagonistic manner to Mic60 as it promotes oligomerization of the F_1_F_O_-ATP synthase and partially restores CJ formation in cells lacking Mic60. Mic10 impairs oligomerization of the F_1_F_O_-ATP synthase similar to Mic60. Applying complexome profiling, we observed that deletion of Mic27 destabilizes the MICOS complex but does not impair formation of a high molecular weight Mic10 subcomplex. Moreover, this Mic10 subcomplex comigrates with the dimeric F_1_F_O_-ATP synthase in a Mic27-independent manner. Further, we observed a chemical crosslink of Mic10 to Mic27 and of Mic10 to the F_1_F_O_-ATP synthase subunit e. We corroborate the physical interaction of the MICOS complex and the F_1_F_O_-ATP synthase. We propose a model in which part of the F_1_F_O_-ATP synthase is linked to the MICOS complex via Mic10 and Mic27 and by that is regulating CJ formation.

## INTRODUCTION

Mitochondria are double-membrane enclosed organelles harboring an outer and an inner membrane. The inner membrane is subdivided into the inner boundary membrane (IBM) which is closely apposed to the outer membrane, and the cristae membrane (CM) which invaginates towards the matrix. The CM is highly variable in size and shape, depending on the tissue and the developmental stage and can show, for instance, tubular, lamellar, or even triangular shapes in electron micrographs 1. Aberrant cristae membrane structures have been associated with numerous severe human diseases 2,3,4, underlining the functional importance of mitochondrial cristae morphology. Cristae are connected to the IBM by cristae junctions (CJs) - highly curved membrane structures with a tubular, ring, or slit-like appearance 5,6,7. They have been proposed to act as diffusion barriers for metabolites and for membrane proteins 7 and to modulate the diffusion of ADP/ATP across the inner membrane 5,8,9,10. This view is supported by reports showing that the inner membrane is subcompartmentalized in a dynamic manner 11,12,13,14. Cristae and CJ remodeling occurs during apoptosis, possibly promoting the efficient release of cytochrome c 15,16,17. In the last years, major progress in understanding the underlying principles of CJ formation has been made. Mic60, termed Fcj1 at that time, was shown to be required for the formation of CJs and also to be localized to CJs in baker’s yeast 18. Mitochondria lacking Mic60 contained concentric stacks of membrane vesicles in their matrix and lacked CJs entirely. Mic60 was later found to be part of a large protein complex with at least five other subunits in baker’s yeast 19,20,21,22. The complex is now termed 'Mitochondrial contact site and cristae organizing system' (MICOS) complex but was also termed MINOS, FCJ1, or MitOS complex before a uniform nomenclature was established 23. The MICOS complex is evolutionarily highly conserved, as orthologues of MICOS subunits exist in mammals, plants and bacteria 24,25. Downregulation of Mic60 (Mitofilin) in mammalian cells led to a massive proliferation of the inner membrane, resulting in a complex network of interconnected membranes and an apparent absence of CJs 26. The other mammalian subunits of the MICOS complex are MIC10/MINOS1 19, MIC13/Qil1 27,28, MIC19/CHCHD3 29, MIC25/CHCHD6 30, MIC26/APOO 31,32, and MIC27/APOOL 33. CHCHD10 was also shown to be a part of the MICOS complex and mutations in CHCHD10 and MIC13 are linked to severe neurological pathologies 34,35. Several interaction partners of mammalian Mic60 were reported, including the coiled-coil-helix-coiled-coil-helix domain proteins 3 and 6 (CHCHD3 and CHCHD6), disrupted-in-schizophrenia 1 (DISC1), the sorting and assembly machinery component 50 (SAM50), metaxin-1 and -2 (MTX1 and MTX2), DNAJC11 19,29,36,37,38, TMEM11 27 and OPA1 29,39. Several subunits of the mammalian MICOS complex have been linked to ageing and to human disorders such as Down's syndrome, Parkinson's disease, and cancer 40,41, underlying the functional importance of CJs and mitochondrial ultrastructure.

The MICOS complex in baker’s yeast is currently known to contain the following other five subunits besides Mic60/Fcj1: Mic10/Mcs1/Mio10/Mos1, Mic12/Aim5/ Fmp51/Mcs12, Mic19/Aim13/Mcs19, Mic26/Mcs29/Mio27, and Mic27/Aim37/Mcs27 41. Mic60 and Mic10 are core subunits of the MICOS complex and lack of those subunits leads to a virtually complete loss of CJs, whereas loss of Mic12, Mic19 or Mic27 have less pronounced and loss of Mic26 has only very minor effects on crista morphology 18,20,21,22,42. The MICOS complex was shown to physically interact with multiple components in the outer (e.g. TOB55/SAM50 complex, Tom40, Ugo1) or the inner membrane (e.g. Mia40) and is functionally linked to the ERMES complex, which links the ER and mitochondria 19,20,21,22,43,44. Our understanding on the role of individual MICOS subunits in shaping the cristae membranes has increased considerably over the last five to eight years [for review see 41]. Next to the central role of Mic60 it became clear that Mic10, which contains unique GXGXGXG motifs, can homooligomerize into a large subcomplex and is able to alter membrane curvature independent of other MICOS subunits 42,45. Mic19 (Mic25 in metazoan) appears to be a regulatory subunit that is peripherally attached to the inner membrane and was proposed to be a redox-sensor of the MICOS complex 46. Mic12, or its putative mammalian paralogue Mic13/Qil1, is required to stabilize the full MICOS complex by holding two subcomplexes, Mic60-Mic19 and Mic10-Mic12/Mic13-Mic26-Mic27, together 27,28,42,47,48,49. Mic26 and Mic27 are homologous proteins, both belonging to the apolipoprotein family which were shown to be linked to the cardiolipin metabolism in mammalian cells 31,32,33. Mic27 in baker’s yeast was proposed to stabilize Mic10 oligomers but, as opposed to Mic12, is not involved in bridging the Mic60-Mic19 to the Mic10-Mic12/Mic13-Mic26-Mic27 subcomplex 48.

Another major mitochondrial complex known to shape cristae membrane curvature is the F_1_F_O_-ATP synthase. It was shown in *Saccharomyces cerevisiae* that deleting the dimer-specific F_1_F_O_-ATP synthase subunits e or g resulted in impaired dimerization and oligomerization of this complex, causing altered, onion-like cristae morphology and a reduced mitochondrial membrane potential 50,51,52,53,54. The three-dimensional arrangement of rows of V-shaped dimers of the F_1_F_O_-ATP synthase is important for generating highly curved membrane rims 14,55,56. We showed that impairing the F_1_F_O_-ATP synthase oligomerization through the deletion of subunits e or g led to altered CJ structure and increased internal cristae branching 18. The same study demonstrated a functional link between the oligomeric state of the F_1_F_O_-ATP synthase and Mic60. We showed that Mic60 and the F_1_F_O_-ATP synthase subunits e or g act antagonistically to control the F_1_F_O_-ATP synthase oligomerization and proposed that thereby membrane curvature is controlled, allowing formation of CJs and cristae rims 18.

In contrast to the F_1_F_O_-ATP synthase and the core MICOS subunit Mic60, the role in CJ formation for other MICOS subunits is not well resolved. Here we focused on the function of the MICOS subunits Mic27 and Mic10. We could show that Mic27 acts in an antagonistic manner to Mic60 and promotes oligomerization of the F_1_F_O_-ATP synthase. Applying complexome profiling, we observed that Mic10 comigrates with the dimeric F_1_F_O_-ATP synthase in a Mic27-independent manner, suggesting that Mic27 is not essential for the assembly of a Mic10 subcomplex. Further, it points to the possibility that a subpopulation of Mic10 interacts with the F_1_F_O_-ATP synthase. The latter is indeed the case, as revealed by chemical crosslinking and coimmunoprecipitation experiments. In sum, we propose a model in which a fraction of the F_1_F_O_-ATP synthase is physically linked to the MICOS complex via Mic10 and Mic27.

## RESULTS 

### Mic27 acts antagonistically to Mic60 and determines crista junction formation

**Figure 1:**
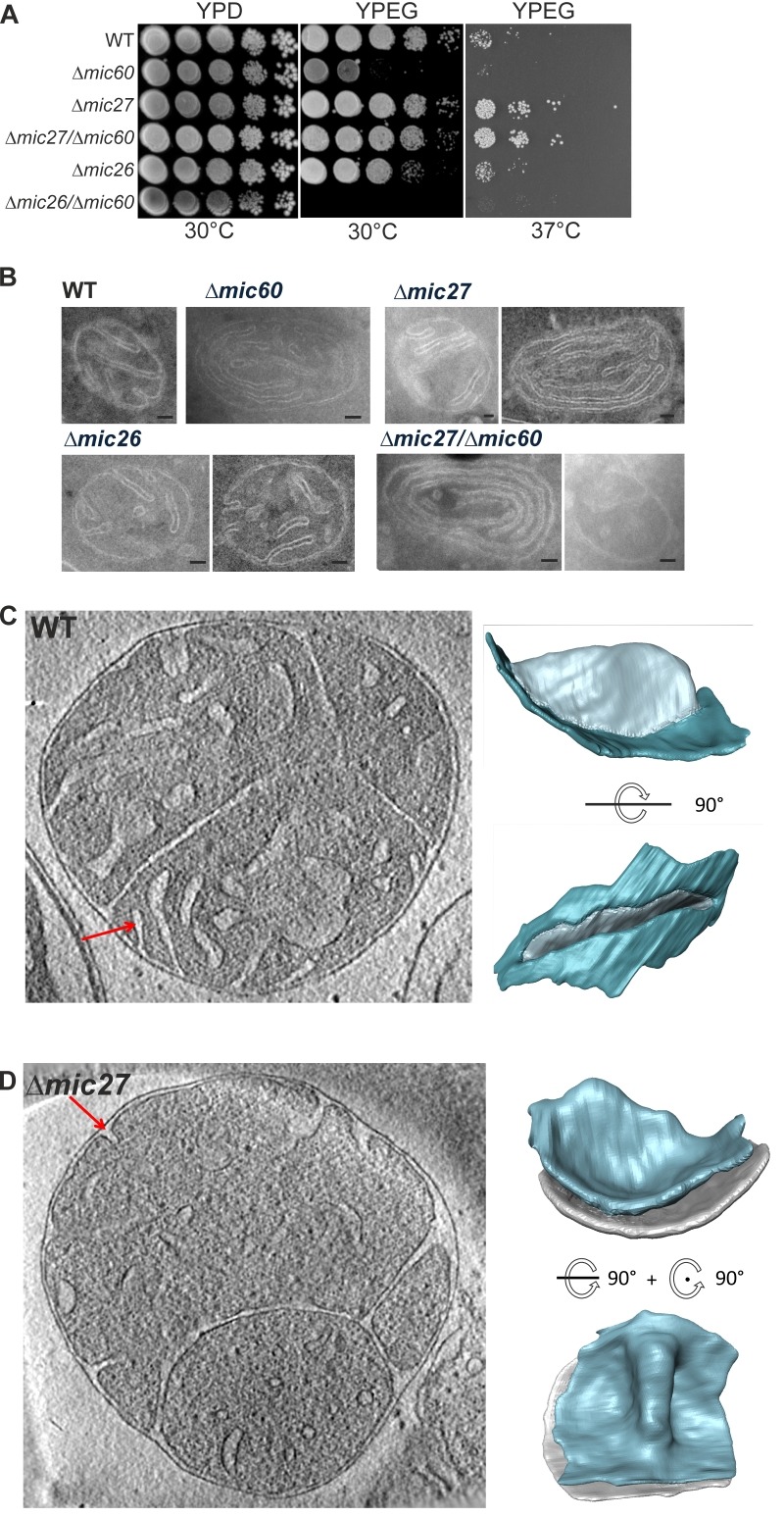
FIGURE 1: Mic27 interacts genetically with Mic60 and deletion of Mic27 leads to altered cristae morphology. **(A)** Growth of WT, Δ*mic26*, Δ*mic27*, Δ*mic60*, Δ*mic27*/Δ*mic60*, and Δ*mic26*/Δ*mic60* strains were examined by serial dilutions on YPD (30°C) and YPEG medium (30 °C and 37°C). **(B)** Indicated strains were cultivated in YPEG, whole cells were chemically fixed and cryo-sectioned, and mitochondrial ultrastructure was determined by standard transmission electron microscopy. Size bars represent 100 nm. **(C,D)** Cryo-electron tomography of isolated wild type (C) and Δ*mic27* (D) mitochondria. Left: slice from a tomogram. Right: surface rendered representation of a crista indicated by red arrow in tomographic slice.

For a better understanding of the role of Mic27 and Mic26 in formation of CJs we determined whether these factors show a genetic interaction with Mic60. For that, we analyzed the growth of wild type, Δ*mic60*, Δ*mic27*, Δ*mic26*, Δ*mic60*/Δ*mic27*, and Δ*mic60*/Δ*mic26* strains, using a drop dilution growth assay on plates containing fermentative (YPD) or non-fermentative (YPEG) growth media at 30°C and 37°C. On a rich medium containing a fermentable carbon source (YPD) we did not observe any difference for those strains (Fig. 1A, left panel). However, we observed a growth defect on a non-fermentable carbon source (YPEG) for the strain lacking Mic60 at both temperatures (Fig. 1A, middle and right panel), and a moderate effect for the Δ*mic26* strain at 30°C when compared to the wild type (Fig. 1A, middle panel). The Δ*mic27* strain, as well as the double-deletion strain Δ*mic60*/Δ*mic27 *did not show a growth defect under respiratory conditions, and even showed a slight growth improvement at 37°C (Fig. 1A, middle and right panel). Thus, at both temperatures, the additional deletion of Mic27 in Δ*mic60* rescued the growth phenotype on YPEG caused by the loss of Mic60. This clearly demonstrates a positive genetic interaction between Mic60 and Mic27, indicating that the loss of Mic27 is epistatic to Mic60 or that Mic60 and Mic27 act in an antagonistic manner. In contrast, for Mic26 we observed a negative genetic interaction with Mic60, as the double-deletion strain Δ*mic60*/Δ*mic26* showed an increased growth defect under respiratory conditions at 30°C compared to Δ*mic60 *(Fig. 1A, middle panel).

Also, at 37°C the additional deletion of Mic26 in Δ*mic60* did not result in a growth rescue on a non-fermentable carbon source (YPEG), which was observed for the additional deletion of Mic27 in Δ*mic60* (Fig. 1A, right panel). This points to opposing functions of Mic26 and Mic27 which appear to act in a synergistic and an antagonistic manner to Mic60, respectively.

Next we determined the mitochondrial ultrastructure of these strains by standard transmission electron microscopy and quantified the number of CJ per mitochondrial section. Consistent with earlier studies 18,20,21,22,44 we observed that cristae morphology is altered in cells lacking Mic60 or Mic27, but not grossly when Mic26 is lacking (Fig. 1B). As expected, the effect was most severe in cells lacking Mic60. The Δ*mic60*/Δ*mic26* strain could not be analyzed in this manner as it did not grow on the non-fermentable carbon source. However, the Δ*mic60*/Δ*mic27* strain exhibited grossly altered cristae morphology which largely resembled the phenotype of the Δ*mic60,* showing many mitochondria lacking crista junctions and internal cristae stacks (Fig. 1B). Still, in the Δ*mic60*/Δ*mic27* strain some mitochondrial sections showed the presence of crista junctions, suggesting a partial restoration of crista junction formation. To quantify this we determined the number of CJs per mitochondrial section in those strains (Table 1).

**Table 1:** Deletion of Mic27 impairs CJ formation but partially restores it in cells lacking Mic60. Indicated strains were grown on non-fermentative growth medium at 30°C, chemically fixed and analyzed by transmission electron microscopy. The number of CJs per mitochondrial section was quantified. The Δ*mic26*/Δ*mic60* was not growing under these growth conditions (see Fig. 1A) and thus could not be analyzed.

**Strain**	**# of mitochondrial sections**	**# of CJs**	**average # of CJs per mitochondrial section**
WT	45	81	1.8
Δ*mic60*	53	0	0.0
Δ*mic27*	77	48	0.62
Δ*mic27*/Δ*mic60*	70	10	0.14
Δ*mic26*	45	78	1.7

The number of CJs per mitochondrial section is reduced in Δ*mic27* cells compared to wild type to about one third (0.6 vs. 1.8). As expected, no CJs were found in Δ*mic60* cells and the abundance of CJs in Δ*mic26* cells was similar to the wild type control. The Δ*mic60*/Δ*mic27* strain showed a very low abundance of CJs (0.14 CJs per mitochondrial section), demonstrating that deletion of Mic27 in cells lacking Mic60 partially restores CJ formation (Table 1). This could serve as a possible explanation for the positive genetic interaction observed between Mic27 and Mic60 shown above (Fig. 1A). Taken together, we conclude that Mic27 is important for CJ formation and is possibly acting in an antagonistic manner to Mic60.

For a more detailed analysis of the mitochondrial ultrastructure in Mic27 deficient cells we performed cryo-electron tomography of isolated mitochondria. We focused on the architecture of cristae rims and CJs. In wild type CJs were found to exhibit a defined shape with narrow slits characterized by sharp edges at the interface between IBM and CM (Fig. 1C). In Mic27-deficient cells CJs appeared quite broad and less defined, showing a more flattened transition from the IBM to the CM, indicating that Mic27 may affect inner mitochondrial membrane curvature (Fig. 1D). Overall, we conclude that Mic27 determines CJ formation and cristae membrane morphology.

### Complexome profiling reveals a destabilization of the MICOS complex upon deletion of Mic27

**Figure 2:**
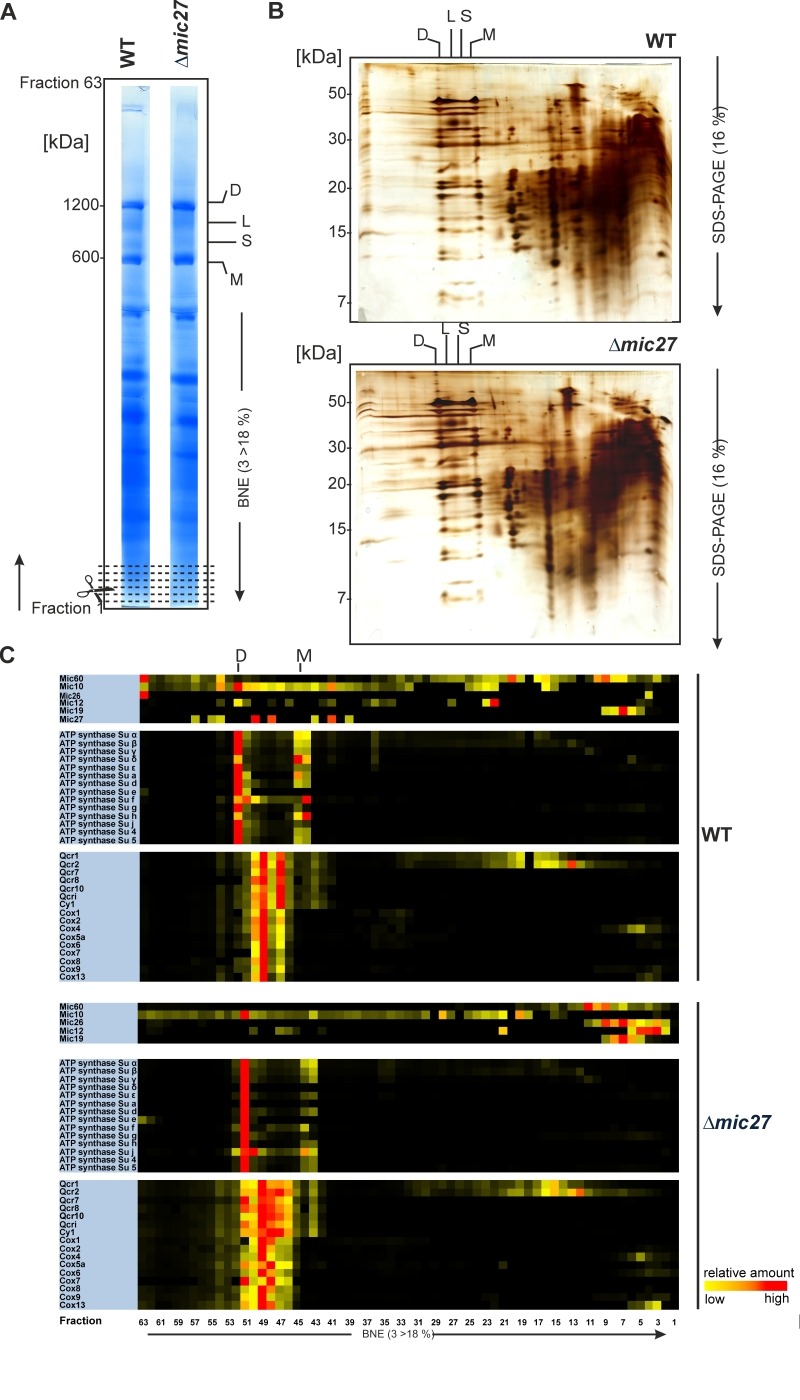
FIGURE 2: Complexome profiling of isolated mitochondria from wild type and Δ*mic27* cells. Mitochondria were solubilized with digitonin (ratio: digitonin to protein 2g/g) and separated with a BN-PAGE (A) or a 2D BN-PAGE/SDS-PAGE was performed (B). 1D BN-PAGE lanes (indicated in panel A) were fixed and stained with Coomassie, sectioned transversely in 63 fractions, and examined by quantitative mass spectrometry. Selected quantified proteins were represented in heat maps (C). The color scale ranges from black (not identified), to yellow (20% of the maximum), to red (maximum abundance). Monomers (M), dimers (D), and oligomers of the F_1_F_O_-ATP synthase (O) are indicated

To investigate the role of Mic27 on the formation of mitochondrial protein complexes, including the MICOS complex, we applied a quantitative complexome profiling approach 57 and compared the migration of native protein complexes in wild type versus Δ*mic27* mitochondria. For that, we isolated mitochondria from these strains, solubilized native mitochondrial protein complexes with digitonin, and separated them by 1D BN-PAGE (Fig. 2A) and by 2D BN/SDS-PAGE (Fig. 2B). After 1D BN-PAGE the gel lane was cut into 63 fractions, all fractions were analyzed by quantitative mass spectrometry, and identified proteins were clustered according to their abundance profile across the 63 fractions (Fig. 2C). The quite abundant mitochondrial complexes involved in oxidative phosphorylation are well resolved and clearly detectable in both wild type and Δ*mic27* mitochondria (Fig. 2AB). This demonstrates that the quality of the mitochondrial preparation as well as the separation of native complexes was good and well comparable between the two samples. To test whether deletion of Mic27 may alter formation or stability of the MICOS complex we directly compared the distribution profiles of these proteins in wild type and Δ*mic27* mitochondria (Fig. 2C). In addition, we show the distribution profiles for numerous subunits representative for Complex III (Cytochrome bc1 complex), Complex IV (Cytochrome c oxidase), and Complex V (F_1_F_O_ ATP synthase). We observed that upon deletion of *MIC27* the MICOS subunits Mic60, Mic26, and Mic12 are not present at a high molecular weight anymore, as opposed to wild type mitochondria (Fig. 2C). These subunits were detected only in fractions at low molecular weight. However, the only MICOS subunit which was still largely present in a fraction at high molecular weight in Δ*mic27* mitochondria was Mic10. This effect of Mic27 on the assembly of the MICOS complex was confirmed by 2D BN/SDS-PAGE and immunoblotting (SFig. 1A). Deletion of Mic27 did not grossly affect the protein levels of Mgm1, Tob55, F_1_F_O_ ATP synthase subunit F1β and subunit e, Tob55) and the MICOS subunits Mic60 and Mic10 (SFig. 1B), suggesting that this effect is not mediated indirectly by any of these proteins. In a recent study deletion of Mic27 also did not lead to an apparent effect on the levels of Mic10 or Mic60 48, consistent with our data. Interestingly, we observed that Mic26 was increased in Δ*mic27* mitochondria and, conversely, also Mic27 in Δ*mic26* mitochondria, again suggesting an antagonistic effect of Mic26 and Mic27. This reciprocal behavior is reminiscent to the situation in mammalian cells 31. Overall, we conclude that Mic27 is important for assembly or stability of the full MICOS complex at high molecular weight, but is not essential for formation of a high molecular weight Mic10 subcomplex.

**Figure 3:**
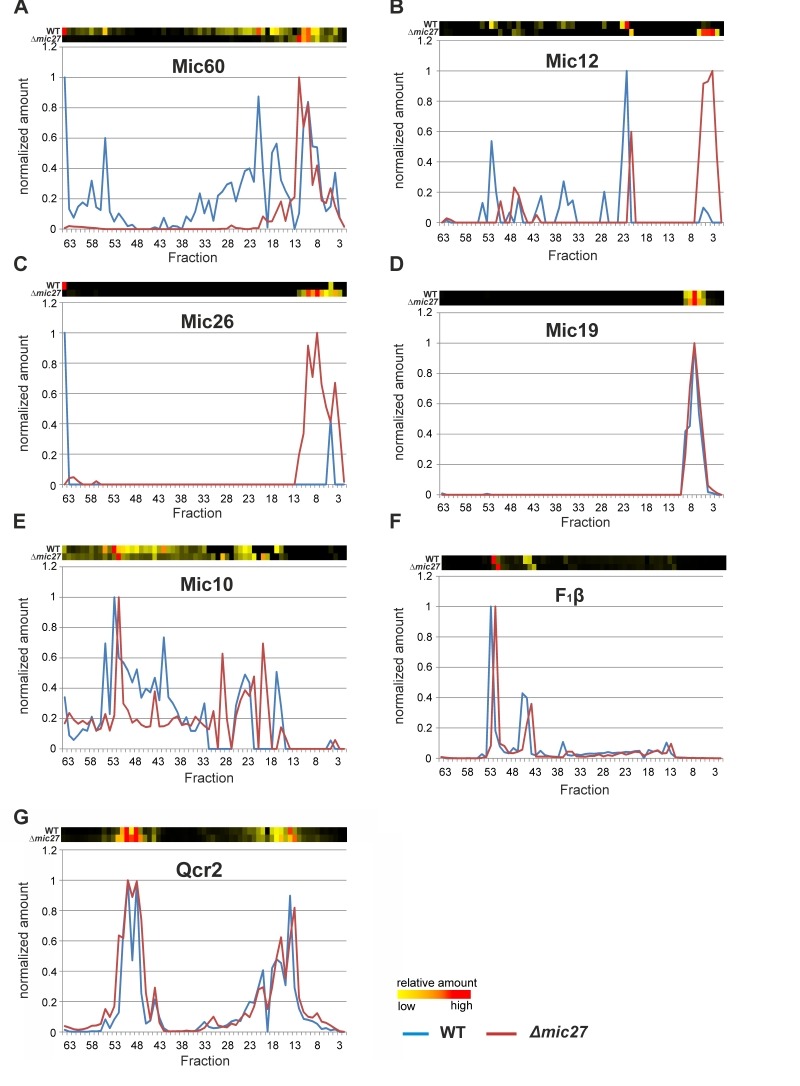
FIGURE 3: Deletion of Mic27 impairs stable assembly of the MICOS complex. **(A-G)** Complexome distribution profiles of Mic60 (A), Mic12 (B), Mic26 (C), Mic19 (D), Mic10 (E), F1β (F), and of Qcr2 (G) of wildtype (WT) and Δ*mic27*-mitochondria (details see Figure 2).

Next, we analyzed the influence of Mic27 on the abundance profile of selected subunits in a more detailed manner (Fig. 3). This different way of representing the data shown in Fig. 2 allows a better direct comparison of individual proteins between the two strains. The observed change from high to low molecular weights for Mic60, Mic12, and Mic26 upon deletion of Mic27 is evident, whereas Mic19 appears at a low molecular weight in both cases (Fig. 3A-D). We attribute the latter to a detergent-induced removal of Mic19 from the MICOS complex even in wild type mitochondria. The size of the high molecular weight complex containing Mic10 appears to be slightly lower as it is shifted by one fraction to the right in Δ*mic27* compared to wild type mitochondria (Fig. 3E). A similar size shift for the dimeric and the monomeric F_1_F_O_ ATP synthase complex but not for Complex III, as indicated by the subunit Qcr2, is observed (Fig. 3FG). The latter observation would argue against an artificial misalignment of individual fractions. Yet, this possibility cannot be excluded for sure, as most subunits of the Complex III run in different fractions, and it is a highly abundant complex, giving a very broad peak which could mask such a size shift. We conclude that Mic27 affects the stability and/or the assembly of the MICOS complex. Future studies will have to address whether lack of Mic27 affects the composition of the high molecular weight Mic10-complex and the F_1_F_O_ ATP synthase.

### The Mic10 subcomplex comigrates with the dimeric F_1_F_O_ ATP synthase independent of Mic27

**Figure 4:**
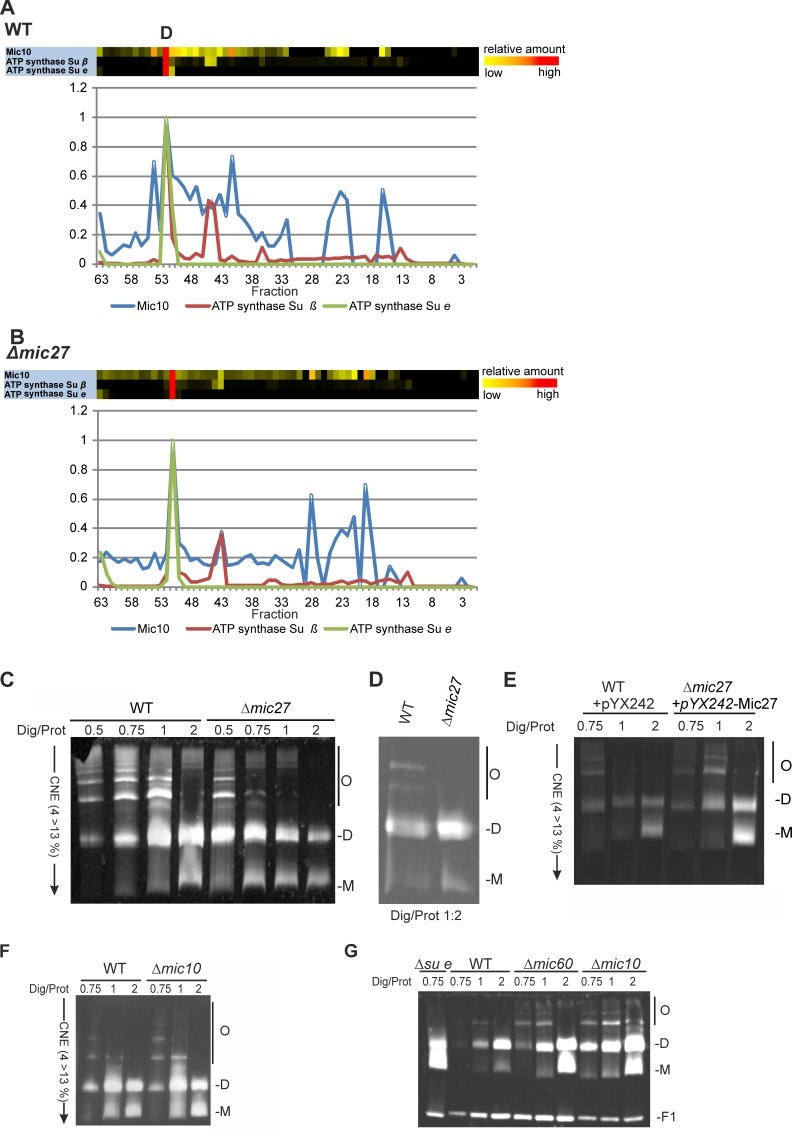
FIGURE 4: Mic27 and Mic10 modulate assembly of F_1_F_O_ ATP synthase oligomers in an antagonistic manner. **(A,B)** Mic10 comigrates with the dimeric F_1_F_O_ ATP synthase irrespective of the presence of Mic27. Complexome distribution profiles of Mic10, F_1_F_O_ ATP synthase subunit β, and the dimer-specific F_1_F_O_ ATP synthase subunit e in wildtype (A) and Δ*mic27* (B) mitochondria (details see Figure 2). **(C-E)** Mic27 promotes the assembly of the F_1_F_O_ ATP synthase oligomers.(C,D) Isolated mitochondria of a wildtype (WT) and a Δ*mic27* yeast strain were solubilized with the indicated ratios of digitonin to protein and separated by CN-PAGE and subsequently in-gel staining of F_1_F_O_-ATP synthase activity was performed. (E) Effect of Mic27 overexpression on assembly of the F_1_F_O _ATP synthase. Indicated strains were analyzed as in panels C,D. Overexpression of Mic27 was confirmed by Western blot analysis (see SFig. 1C,). **(F,G)** Mic10 moderately impairs the assembly of the F_1_F_O_ ATP synthase oligomers. Indicated strains were analyzed as in panels C,D. Strains lacking the F_1_F_O_ ATP synthase subunit e or Mic60 were used as controls in panel G. Monomers (M), dimers (D), oligomers of the F_1_F_O_-ATP synthase (O), and the F1 part (F1) of the F_1_F_O_ ATP synthase are indicated.

The possible effect of Mic27 deletion on Mic10 and the F_1_F_O_ ATP synthase prompted us to analyze this further. A direct comparison of Mic10 and two subunits of the F_1_F_O_ ATP synthase revealed that the Mic10-containing subcomplex actually comigrates with the dimeric F_1_F_O_ ATP synthase both in wild type and in Δ*mic27* mitochondria (Fig. 4AB). A partial, less pronounced comigration of Mic10 is also observed with the monomeric F_1_F_O_ ATP synthase in both types of mitochondria. Taken together, both the Mic10 subcomplex and the dimeric F_1_F_O_ ATP synthase migrate to a major extent in the same fraction in BN-PAGE, suggesting a possible functional interplay between the MICOS complex and the F_1_F_O_ ATP synthase.

This comigration is independent of Mic27, suggesting that Mic10 may be able to form a high molecular weight subcomplex lacking Mic27, which is associated with the F_1_F_O_ ATP synthase.

### Mic10 and Mic27 have opposing effects on the oligomerization of the F_1_F_O-_ATP synthase

For Mic60 we have shown earlier that it impairs dimerization and oligomerization of the F_1_F_O_ ATP synthase in *S. cerevisiae* 18. Thus, we decided to analyze whether Mic27 or Mic10 modulate the dimerization and oligomerization of the F_1_F_O_ ATP synthase, using CN-PAGE and subsequent in-gel staining of the F_1_F_O_-ATP synthase activity. We observed that deletion of Mic27 led to reduced amounts of oligomeric F_1_F_O_ ATP synthase (O) and to increased amounts of monomeric F_1_F_O_ ATP synthase (M) complexes when compared to wild type control mitochondria (Fig. 4C and D). This was clearly evident at different ratios of digitonin to protein but is particularly well seen at a ratio of 0.75 and 1 (Fig. 4C). On the contrary, overexpression of Mic27 promoted oligomerization of the F_1_F_O_ ATP synthase (Fig. 4E). The successful overexpression of Mic27 was validated by immunoblotting (SFig. 1C). We conclude that Mic27 promotes oligomerization of the F_1_F_O_ ATP synthase und thus acts in an antagonistic manner to Mic60, consistent with the results described above (Fig. 1A and B and Table 1). Next we checked how deletion of Mic10 affects dimerization and oligomerization of the F_1_F_O_ ATP synthase. We observed that Mic10 moderately impairs this process and thus acts in an antagonistic manner to Mic27 but in a similar way as Mic60 (Fig. 4FG). Taken together, we could identify a functional, yet reciprocal, interplay of Mic27 and Mic10 modulating the oligomerization of the F_1_F_O_ ATP synthase.

### Mic10 physically links the dimeric F_1_F_O_ ATP synthase to Mic27

**Figure 5:**
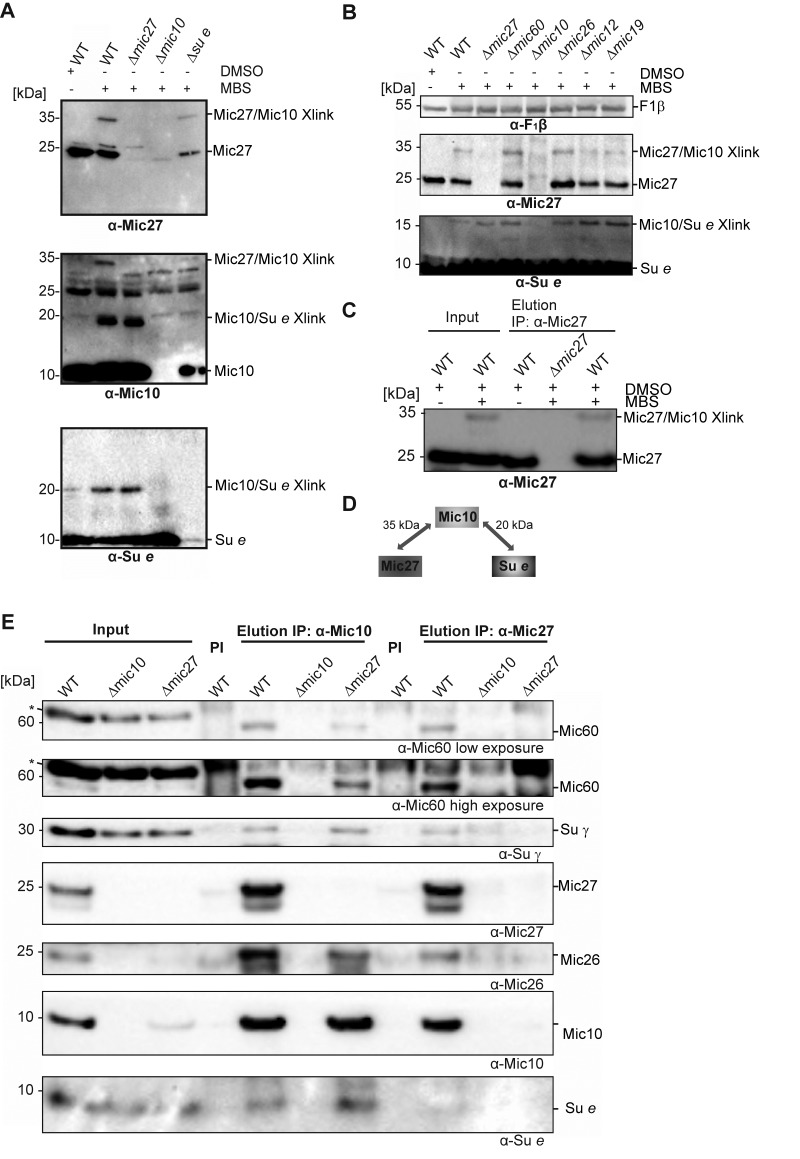
FIGURE 5: Mic10 is physically linked to the F_1_F_O_-ATP synthase. **(A)** Isolated wildtype- (WT), Δ*mic27*-, Δ*mic10*- and Δ*su e*-mitochondria were treated with the chemical crosslinker MBS (m-maleimidobenzoyl-N-hydroxysuccinimideester). Wild type mitochondria treated with DMSO served as a negative control. Crosslinked proteins were analyzed by SDS-PAGE and Western blotting. A crosslink of Mic27 and Mic10 (Mic27/Mic10) was identified at 35 kDa and a crosslink of Mic10 and Su e (Mic10/Su e) at 20 kDa. **(B)** Chemical crosslinking was performed with isolated mitochondria of indicated yeast strains: wild type (WT), Δ*mic27*, Δ*mic60*, Δ*mic10*, Δ*mic26*, Δ*mic12* and Δ*mic19*. **(C)** Immunoprecipitation of Mic27-Mic10 crosslink. Isolated wild type- (WT) or Δ*mic27*-mitochondria were treated or not with the crosslinker MBS, solubilized with digitonin (4 g/g) and co-immunoprecipitation using a purified anti-Mic27 (right part) antibody was performed. As negative controls, mitochondria not treated with MBS and the corresponding deletion strain were used. The asterisk (*) indicates a nonspecific band of the anti-Mic60 antibody. **(D)** Schematic representation of the described chemical crosslinks of Mic10 with Mic27 and Su e. **(E)** Isolated wild type- (WT), Δ*mic10*- and Δ*mic27*-mitochondria were solubilized with digitonin (4g/g) and co-immunoprecipitation using a purified anti-Mic10 (middle part) or anti-Mic27 (right part) antibody was performed. As negative controls, mitochondria treated with the corresponding pre-immune serum (PI) and the corresponding deletion strains were used. The asterisk (*) indicates a nonspecific band of the anti-Mic60 antibody.

Based on these findings we asked whether there is a physical interaction between the MICOS complex and the F_1_F_O_ ATP synthase. To test such possible interaction we treated isolated wild type mitochondria with the chemical crosslinker MBS (m-maleimidobenzoyl-N-hydroxysuccinimide ester) and analyzed the crosslinks by Western Blot analysis. DMSO-treated mitochondria as well as mitochondria isolated from indicated deletion strains were used as negative controls. We could observe a specific chemical crosslink at ~35 kDa using antibodies raised against Mic27 as well as Mic10 (Fig. 5A, top and middle panel; Fig. 5B). This 35 kDa-band was absent in the DMSO control and in mitochondria lacking Mic10 as well as Mic27 consistent with the interpretation that this band represents a Mic27-Mic10 adduct. To validate this further we also performed a chemical crosslinking experiment followed by immunoprecipitation using affinity purified anti-Mic27 antibodies (Fig. 5C). The 35 kDa-band was eluted in a MBS- and Mic27-dependent manner, confirming that the crosslink contains Mic27. Based on these findings and on the size we conclude that the 35 kDa band is indeed the Mic27-Mic10 adduct.

Moreover, we detected a chemical crosslink at the size of ~20 kDa when using antibodies raised against Mic10 and the F_1_F_O_ ATP synthase subunit e (Fig. 5A, middle and bottom panel; Fig. 5B). This crosslink product was absent in the DMSO control and when mitochondria were isolated from Δ*mic10* and Δ*su e* cells. Based on this and the size of the crosslink we conclude that the 20 kDa-band indeed represents a Mic10-Su e adduct. Interestingly, the crosslink was still present in Δ*mic27* mitochondria, indicating that Mic10 interacts with F_1_F_O_ ATP synthase subunit e in a Mic27-independent manner. This is consistent with the fact that the Mic10 subcomplex comigrates with the F_1_F_O_ ATP synthase also when Mic27 is lacking (Fig. 4B). Overall, we could show that Mic10 is in very close local proximity to Mic27 as well as to the F_1_F_O_-ATP synthase subunit e (Fig. 5D), suggesting a physical interaction between the MICOS complex and the F_1_F_O_-ATP synthase. We aimed to corroborate this idea by a co-immunoprecipitation experiment with affinity purified anti-Mic10 and anti-Mic27 antibodies. We observed a physical interaction of Mic10 with several MICOS subunits and with the F_1_F_O_-ATP synthase subunit γ and the F_1_F_O_-ATP synthase subunit e (Fig. 5E). These interactions are specific as they are absent in the negative control using Δ*mic10* mitochondria. In Δ*mic27* mitochondria the interaction of Mic10 with the F_1_F_O_-ATP synthase is still present, even slightly more pronounced, confirming our earlier notion that part of Mic10 remains associated to the F_1_F_O_-ATP synthase, even when Mic27 is absent. At the same time the interaction of Mic10 is markedly reduced with Mic60 or Mic26 in Δ*mic27* mitochondria, which is in line with the destabilization of the MICOS complex in these mitochondria. Another explanation could be the unexpected low level of Mic10 observed in the input fraction derived from Δ*mic27* mitochondria. However, this we attribute to a blotting problem since Mic10 was co-immunoprecipitated from Δ*mic27* mitochondria as efficiently as from wild type mitochondria when using the anti-Mic10 antibody (Fig. 5E, lanes 5 vs. 7). Moreover, Mic10 is consistently not grossly affected in Δ*mic27* mitochondria (SFig. 1B and C and see ref. 47). Using the anti-Mic27 antibody, we could confirm the interaction with F_1_F_O_-ATP synthase subunit γ but not with F_1_F_O_-ATP synthase subunit e consistent with the observed crosslink of Su e to Mic10 but not to Mic27. Taken together, we propose that Mic10 is partially associated to the dimeric F_1_F_O_-ATP synthase, presumably via subunit e, and that this occurs largely in a Mic27-independent manner.

## DISCUSSION 

**Figure 6:**
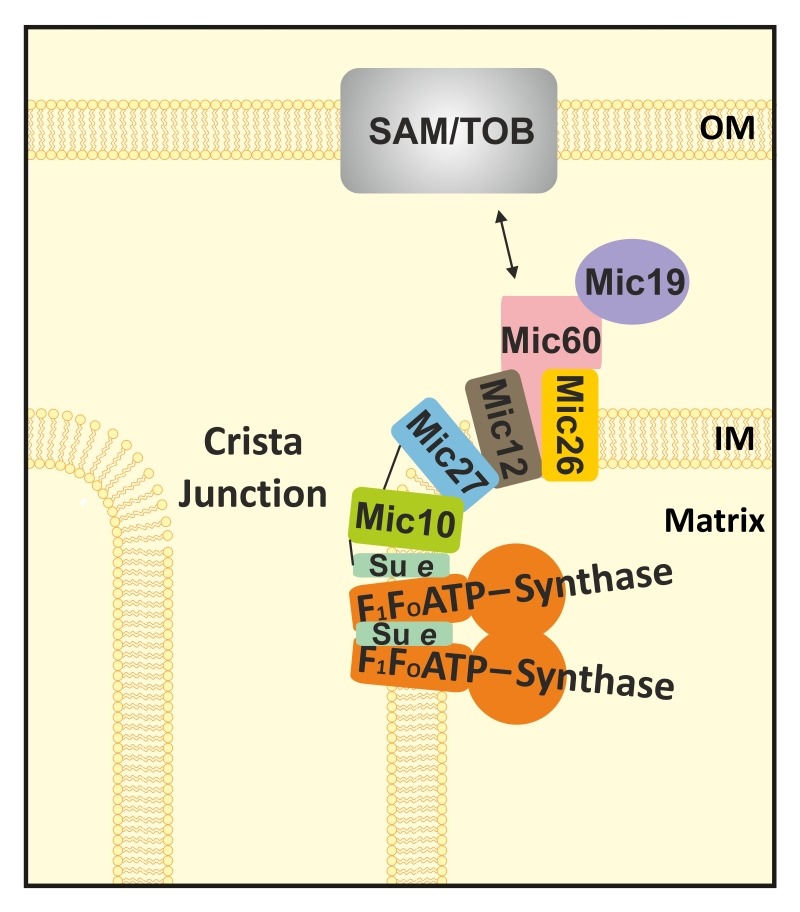
FIGURE 6: Model for the functional interplay between the MICOS-complex and the F_1_F_O_-ATP synthase in yeast. Schematic representation of MICOS subunits in the mitochondrial inner membrane. Mic60 (pink) interacts (black arrow) via its C-terminus with protein complexes (grey) in the outer membrane (OM; e.g. the SAM-/TOB-complex). Mic27 (blue) mediates the interaction between Mic10 (green) and the remaining MICOS-complex. Mic10 interacts with part of the F_1_F_O_-ATP synthase (orange) via the dimer-specific F_1_F_O_-ATP synthase subunit e (Su e in pale green). Black lines indicate observed crosslinks. OM: outer membrane, IM: inner membrane.

There are numerous factors known to determine the architecture of the mitochondrial inner membrane in a direct or indirect manner 1. Mitochondrial ultrastructure is altered by e.g. impairing the function of numerous factors, by alterations in metabolism, upon induction of apoptosis, and in many examples of human disorders linked to different kinds of mitochondrial dysfunction. Two complexes that have turned out to be central in modulating cristae membrane structure in a rather direct manner are the F_1_F_O_-ATP synthase and the MICOS complex. The latter is well known to be enriched at CJs and to be essential for CJ formation. The role of the two core subunits, Mic60 and Mic10, is studied to some extent, yet the other MICOS subunits are less well studied [for review see 41]. For Mic10, its ability to bend membranes *in vitro *has been demonstrated 45. The F_1_F_O_-ATP synthase is well known for its fundamental bioenergetic role in oxidative phosphorylation. In addition, it has been shown to be able to form dimers and oligomers which can bend the inner membrane, forming cristae tips or rims 14,50,51,52,53,54,55,56. The dimerization and higher order arrangement into oligomers depend on the dimer-specific F_1_F_O_-ATP synthase subunits e and g, as shown for *S. cerevisiae* 50,53,54. Lack of these subunits was indeed shown to cause an increase in the number of CJs per mitochondrial section, a decrease in number of cristae tips per mitochondrial section, and an increase in CJ diameter 18. Moreover, this study demonstrated that the F_1_F_O_-ATP synthase subunits e and g act in an antagonistic manner to Mic60/Fcj1, pointing to a functional interplay of the F_1_F_O_-ATP synthase with the MICOS complex. Here we provide insight into how these two complexes interact functionally. The following observations underscore such an interplay. First, a subpopulation of Mic10 was found to comigrate with the F_1_F_O_-ATP synthase in wild type as well as in Δ*mic27* mitochondria. Second, deletion of Mic27 impaired dimerization and oligomerization of the F_1_F_O_-ATP synthase, indicating that Mic27 promotes formation of higher oligomers of this complex. Third, deletion of Mic10 promoted dimerization and oligomerization of the F_1_F_O_-ATP synthase, thus, Mic10 may act in a similar manner as Mic60 18. Fourth, chemical crosslinking of Mic10 to the F_1_F_O_-ATP synthase subunit e was observed. Fifth, coimmunoprecipitation experiments revealed a physical interaction between the F_1_F_O_-ATP synthase and the MICOS complex. During the preparation of this manuscript an independent study appeared, reporting also a physical interaction between the MICOS complex and the F_1_F_O_-ATP synthase 58. Overall, we can conclude that in the absence of Mic27, when most of the high-molecular weight MICOS complex is destabilized, a Mic10 subcomplex remains associated with the F_1_F_O_-ATP synthase, suggesting that Mic27 is important to bridge a Mic10/F_1_F_O_-ATP synthase subcomplex to the remaining Mic60-Mic19-Mic27-Mic26-Mic12 subcomplex (see model depicted in Fig. 6). This is also supported by the crosslink of Mic10 to Mic27 (Fig. 5A-D) and by the fact that much less Mic60 and Mic26, but slightly more F_1_F_O_-ATP synthase, is bound to Mic10 in Δ*mic27* mitochondria compared to wild type mitochondria (Fig. 5E). Mic12 was earlier shown to bridge the Mic60-Mic19 and the Mic10-Mic12-Mic26-Mic27 subcomplexes in baker’s yeast 48. The importance of Mic12, or its putative metazoan orthologue Mic13/Qil1, in this process was well supported 27,28,42,47,48,49. However, it should be noted that Mic27 levels are strongly reduced in strains lacking Mic12 or Mic10 (SFig. 1B), consistent with earlier studies 20,21,22,42,45. Based on these findings and our study here we propose that next to Mic12, Mic27 has a major role in bridging the Mic60-containing subcomplex with the Mic10-containing subcomplex. This study shows that the latter subcomplex is partly associated with the F_1_F_O_-ATP synthase, even upon deletion of Mic27. The role of Mic27 in bridging two MICOS subcomplexes does not exclude the other proposed role for this MICOS subunit, namely to stabilize Mic10 homooligomers and modulate Mic10-dependent membrane curvature 48. The crosslink of Mic10 to the F_1_F_O_-ATP synthase subunit e suggests that these small subunits can bind to each other and thus mediate and modulate directly the interaction between the MICOS complex and the F_1_F_O_-ATP synthase.

Although our study does not allow solid conclusions on the stoichiometry of the composition of the subcomplexes, we see that Mic10, and to a minor extent also Mic12, are the only MICOS subunits that comigrate partially with the dimeric F_1_F_O_-ATP synthase in wild type and Δ*mic27* mitochondria. A partial - as opposed to a complete - overlap is expected as the F_1_F_O_-ATP synthase is highly abundant in mitochondria and believed to be enriched in cristae tips or cristae rims rather than being close to CJs 11,18,56. What could be the role of this partial interaction? We propose the following model (Fig. 6). There is a physical connection of a fraction of the F_1_F_O_-ATP synthase located near CJs to the MICOS complex. This occurs possibly in a ring-or slit-like fashion which would be well compatible with tubular cristae extensions near a particular CJ. This would well account for the reported size variations in the lengths of CJs from 30 to 200 nm 1,5,6,7 and the idea that also tubular cristae at the neck contain oligomers of F_1_F_O_-ATP synthase 18. Future studies will have to decipher how this interaction is regulated and how it modulates cristae morphology under different metabolic conditions.

## MATERIALS AND METHODS

### Strains and plasmids

The cultivation of yeast cells was carried out at 30°C as described 59. YPEG or YPD were used as culture media if not stated differently. Strains containing a plasmid were cultivated in appropriate selective media for selection of auxotrophy markers.

### Recombinant DNA techniques

In the haploid strain Δ*mic60*/Δ*mic27* (BY4742 background) the open reading frame of *MIC60* was deleted in the strain Δ*mic27* (BY4742) by homologous recombination of a deletion cassette of the Mic60 locus as described previously 18. To overexpress Mic27, the open reading frame (ORF) of *MIC27* was cloned in pYX242, using restriction sites *Hind*III and *Sac*I. The ORF encoding Mic27 was generated by PCR from genomic wild type DNA (W303α) with the oligonucleotides Mic27fw (5’- CCC CCC AAG CTT ATG GTA AAT TTT TAT GAT GAC GTG G -3’) and Mic27rev (5’ CCC CCC GAG CTC TCA TGC TTG CTC CAA CTT TTC ATA AAG C -3’).

### Analysis of growth phenotype

To determine the growth, drop dilution assays were performed. For that the same number of yeast cells from exponentially growing liquid yeast cultures were taken, diluted in 1:5 (30°C) or 1:10 (37°C) steps, and equal volumes were transferred to YPD or YPEG plates. After two to three days at 30 °C or 5 to 7 days at 37°C growth was evaluated.

### Isolation of mitochondria

Yeast strains were cultured under exponential growth conditions in nonfermentable medium (YPEG or selective SG medium supplemented with 0.1% glucose) for at least 3 days at 30°C. Cells were harvested by centrifugation (5 min at 1,500 g), washed with water, treated with 2 ml of DTT buffer (10 mM DTT, 100 mM Tris/SO_4_ pH 9.4) per 1 g of wet weight, shaked at 150 rpm for 10 - 20 min at 30°C, and centrifuged for 5 min at 1,500 g. Next, cells were washed with sorbitol buffer (1.2 M sorbitol, 20 mM KPi, pH 7.4) and treated with zymolyase (3-6 mg per 1 g wet weight), shaked at 150 rpm for 1 h at 30°C to generate spheroplasts, washed, resuspended in NMIB buffer (20 mM HEPES pH 7.4, 0.6 M sorbitol, 5 mM MgCl_2_, 50 mM KCl, 100 mM KOAc, 1mM PMSF) and homogenized in a glass homogenizer. The obtained total cell extract was centrifuged at 3,000 g for 5 min to remove cell debris and the supernatant was centrifuged at 10,250 g for 10 min to separate crude mitochondria (pellet) and cytosol (supernatant). The isolated crude mitochondria were resuspended in NMIB buffer at 10 mg/ml total protein concentration, frozen in liquid nitrogen and stored at -80°C.

### Electron microscopy and cryo electron tomography

Electron microscopy using the Tokuyasu method was done as described 11. Cryopreparation and cryo-EM tomography of isolated mitochondria from indicated strains was carried out as described 55.

### BN- and CN-PAGE

Blue native polyacrylamide gel electrophoresis (BN-PAGE) to separate native protein complexes according to size was done as described 60,61. In brief, 100-400 μg of isolated and solubilized mitochondria were applied to gradient gels together with Serva Blue G or without the dye for CN-PAGE 57. Gels were stained with Coomassie or directly used for an in-gel ATPase activity assay (see below).

### Complexome Analysis

Complexome profiling analysis was applied to determine the quantitative distribution of subunits in native protein complexes after BN-PAGE. It was essentially done as described 62. In brief, 100 μg mitochondria were solubilized with digitonin and the protein complexes were separated by native electrophoresis. Gels were stained with Coomassie and lanes were cut from bottom to top into 63 equal fractions. The mass spectrometric analysis was performed using an LTQ / Orbitrap XL mass spectrometer (Thermo Fisher Scientific) equipped by a nano Agilent1200 HPLC. The raw data were analyzed by MaxQuant 1.5.0.30 63, the peptides and protein identification was performed with the reference proteome set of *S. cerevisiae* (version January, 2015 by Uniprot.org, with 6741 entries). The data were analyzed by NOVA software 64.

### In-gel ATPase activity assay

The native complexes of the F_1_F_O_-ATP synthase were visualized in CN-PAGE gels as described 65. After electrophoresis, the gels are incubated in 0.2% (w/v) Pb(NO_3_)_2_; 14 mM MgCl_2_; 8 mM ATP; 35 mM Tris/HCl pH 8.3; 270 mM Glycine/NaOH pH 8.3 for up to 12 h.

### Chemical crosslinking

For crosslinking, isolated mitochondria were resuspended in 0.6 M Sorbitol; 20 mM HEPES pH 7.4 and incubated with 400 μM MBS for 30 minutes. As a negative control a sample was used only with DMSO. The reaction was stopped with 100 mM glycine, pH 8.

### Immunoprecipitation

Corresponding affinity purified antibody was coupled to a protein A Sepharose CL-4B matrix (GE Healthcare Life Sciences). Mitochondria were solubilized in 50 mM NaCl; 50 mM Imidazol/HCl pH 7, 0.2 mM Aminocarpoic acid, 1 mM EDTA; Complete (Roche); 1 mM PMSF; 0.5 mM Phenanthroline; 2 μg/ml Aprotinin; 1 μg/ml Pepstatine and solubilized with 4 g/g of digitonin. The bound material was eluted at 65°C using Laemmli SDS loading buffer and analyzed by Western blot analysis. Antibodies against Mic27 or Mic10 were raised in rabbits against the peptides NH2-CIRYAREQLYEKLEQA-COOH (aa 219 to 233) and NH2-CEGDAIFRSSAGLRSSKV-COOH for Mic10 (aa 80 to 96), respectively (Pineda, Germany). For Mic27, the same peptide was also used for raising an antibody in chicken (Dabio, Germany) and was used for affinity purification. Antibodies against Mic26 were kindly provided by Nikolaus Pfanner and Martin van der Laan. Other antibodies were obtained earlier as described 11,18,50,66.

## SUPPLEMENTAL MATERIAL

Click here for supplemental data file.

All supplemental data for this article are also available online at http://microbialcell.com/researcharticles/cristae-architecture-is-determined-by-an-interplay-of-the-micos-complex-and-the-f1fo-atp-synthase-via-mic27-and-mic10.
