# Supplementary material for: Cristae architecture is determined by an interplay of the MICOS complex and the F1FO ATP synthase via Mic27 and Mic10
Source: Microb Cell. 2017 Jul 20;4(8):259–72. doi: 10.15698/mic2017.08.585 (PMC5568431; doi:10.15698/mic2017.08.585)
Supplement: Supplementary file 1 [file mic-04-259-s01.pdf]

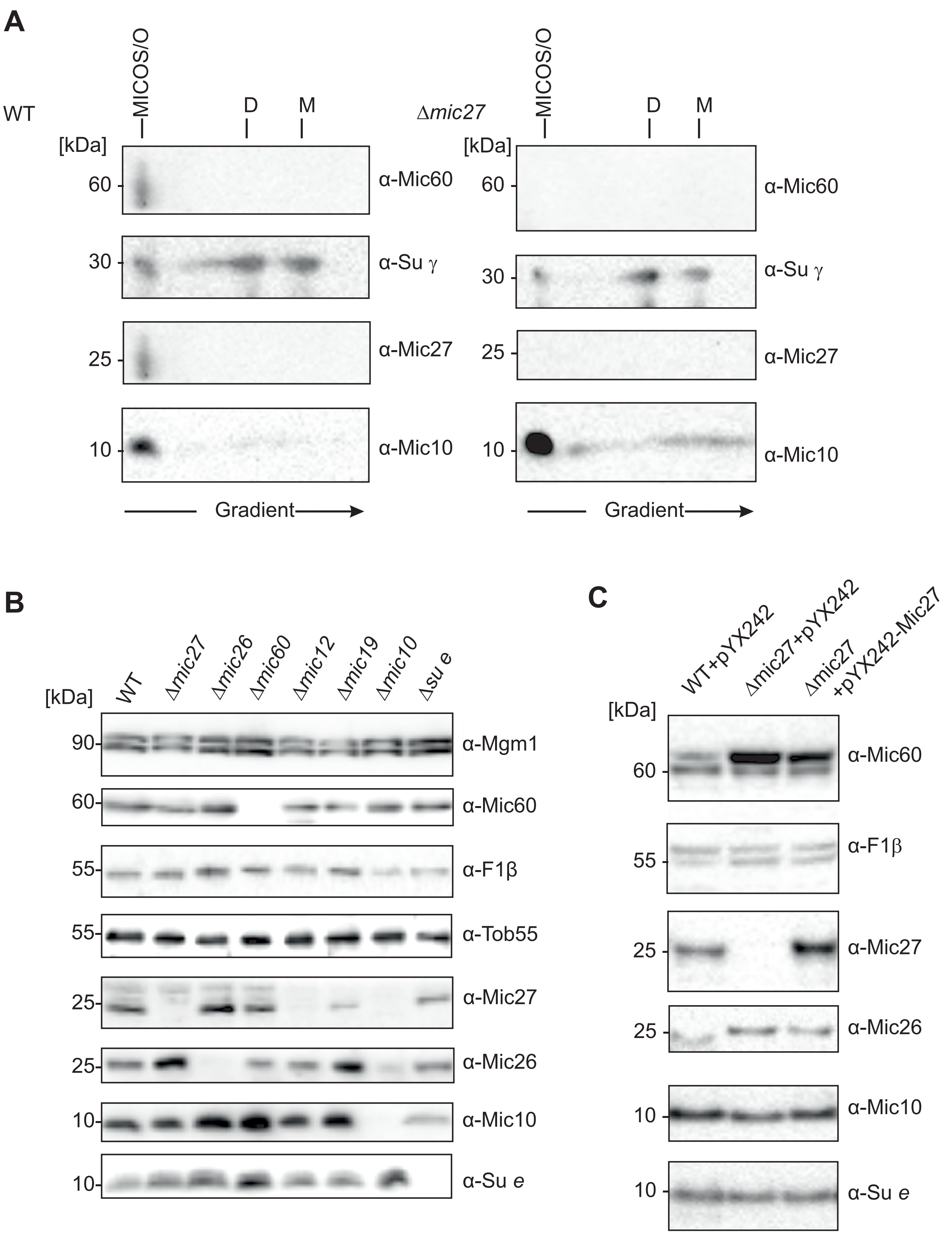

Supplemental Figure 1

Supplemental figure legends

Supplemental Figure 1. Mic27 is required to stabilize the MICOS complex but not for formation of a high molecular weight complex containing Mic10. A, 2D BN-PAGE. Isolated mitochondria of a wildtype and a *Δmic27* yeast strain were solubilized with digitonin (ratio: digitonin to protein 2g/g) and separated by BN-PAGE (1st dimension) and excised gel strips were used for Tris-tricine SDS-PAGE (2nd dimension), and western blot analysis was performed. Monomers (M), dimers (D), and oligomers of the F_1F_O-ATP synthase (O) are indicated. **B**, Mic27 and other subunits were tested for their expression in MICOS deletion mutant strains and in *Δsu e* cells by western blot analysis. Indicated mitochondrial marker proteins were analyzed, Mgm1, Sam50/Tob55, F1β and Su *e*, and subunits of the MICOS-complex, Mic60, Mic10, Mic26, and Mic27. **C,** Validation of Mic27 overexpression using a *Δmic27* strain transformed with pYX242-Mic27. Mic27 and other subunits were tested for their expression in the indicated strains. A wild type and a *Δmic27* yeast strain harboring the empty vector (pYX242) were used as controls. Indicated mitochondrial marker proteins were analyzed, F1*β* and Su *e* and subunits of the MICOS-complex: Mic60, Mic10, Mic26, and Mic27, using western blot analysis.
